# Primary adrenal insufficiency due to bilateral adrenal hemorrhage-adrenal infarction in a patient with systemic lupus erythematosus and antiphospholipid syndrome: case presentation and review of the literature

**DOI:** 10.1007/s42000-023-00463-5

**Published:** 2023-07-12

**Authors:** K. Bouki, V. Venetsanaki, M. Chrysoulaki, A. Pateromichelaki, G. Betsi, V. Daraki, N. Sbyrakis, K. Spanakis, G. Bertsias, P. I. Sidiropoulos, Paraskevi Xekouki

**Affiliations:** 1 Endocrinology and Diabetes Clinic, University Hospital of Heraklion, University of Crete School of Medicine, Voutes, 71500 Heraklion Crete, Crete Greece; 2 Rheumatology and Clinical Immunology, University General Hospital of Heraklion, Crete, Greece; 3 Emergency Department, University General Hospital of Heraklion, Crete, Greece; 4 Interventional Radiology Unit, Department of Medical Imaging, University General Hospital of Heraklion, Crete, Greece

**Keywords:** Adrenal insufficiency, Adrenal failure, Hypoadrenalism, Addison’s disease, Adrenal hemorrhage, Antiphospholipid syndrome, Systemic lupus erythematosus

## Abstract

Primary adrenal insufficiency (PAI) is a rare disease which represents the end stage of a destructive process involving the adrenal cortex. Occasionally it may be caused by bilateral adrenal hemorrhagic infarction in patients with antiphospholipid syndrome (APS). We herein report the challenging case of a 30-year-old female patient with systemic lupus erythematosus (SLE) and secondary APS who was admitted to the emergency department (ED) due to fever, lethargy, and syncopal episodes. Hyponatremia, hyperkalemia, hyperpigmentation, shock, altered mental status, and clinical response to glucocorticoid administration were features highly suggestive of an acute adrenal crisis. The patient’s clinical status required admission to the intensive care unit (ICU), where steroid replacement, anticoagulation, and supportive therapy were provided, with a good outcome. Imaging demonstrated bilateral adrenal enlargement attributed to recent adrenal hemorrhage. This case highlights the fact that bilateral adrenal vein thrombosis and subsequent hemorrhage can be part of the thromboembolic complications seen in both primary and secondary APS and which, if misdiagnosed, may lead to a life-threatening adrenal crisis. High clinical suspicion is required for its prompt diagnosis and management. A literature search of past clinical cases with adrenal insufficiency (AI) in the setting of APS and SLE was conducted using major electronic databases. Our aim was to retrieve information about the pathophysiology, diagnosis, and management of similar conditions.

## Introduction

Primary adrenal insufficiency (PAI) is a rare disease which is caused by the destruction of the adrenal cortex, leading to deficient secretion of glucocorticoids, mineralocorticoids, and androgens [1, 2]. It was first described by Thomas Addison in 1855 and is therefore frequently termed Addison’s disease [3]. It becomes clinically apparent when more than 90% of both adrenal cortices is destroyed [2]. PAI was associated with very short life expectancy until the synthesis of cortisone by Kendall, Sarett, and Reichstein and the introduction of glucocorticoid replacement treatment in 1948 [4–6].

Recent epidemiological studies indicate a rising prevalence of PAI. This is attributable to the growing number of chronically and severely ill patients who require intensive care with multiple pharmacological therapies and to additional iatrogenic factors such as immune checkpoint inhibitors, anticoagulants, and drugs which affect cortisol synthesis, action, and metabolism. Moreover, improvement in diagnostic methods and better reporting also contribute to the increasing manifestation of PAI [7].

The highest prevalence is documented in Nordic countries at 15–22 individuals per 100,000 population, [8–11] whereas other European countries report 10 cases per 100,000 [12–14]. Addison’s disease is usually diagnosed in the third to fifth decades [15]. Regarding etiology, autoimmune adrenalitis is the most common cause of PAI in adults, accounting for up to 90% of cases in Western countries [7]. It can be either isolated (40%) or a component of autoimmune polyglandular syndromes (60%) [16].

Infections, mainly tuberculosis, are another prominent cause of PAI. Genetic disorders, more likely to be diagnosed in children, are represented by congenital adrenal hyperplasia (72%), autoimmune polyglandular syndrome type 1, adrenoleukodystrophy, and congenital adrenal hypoplasia [17]. Unusual causes of PAI are metastatic adrenal diseases, lymphomas, infiltrative disorders, bilateral adrenalectomy, and drugs [2].

PAI caused by bilateral adrenal hemorrhage or infarction is rare but possibly fatal. It is well known to be associated with meningococcemia (Waterhouse-Friderichsen syndrome) or pseudomonas aeruginosa infection. It may also be encountered in severe traumas, disseminated intravascular coagulopathy, antiphospholipid syndrome (APS) or other thrombophilic conditions, bleeding disorders, and use of anticoagulants [18].

The etiologies are diverse. However, the present report focuses on PAI in the setting of APS. APS, first described in 1983, is an autoimmune disorder characterized by hypercoagulability, potentially resulting in thrombosis of all segments of the vascular system. It is diagnosed in less than 0.5% of all patients presenting with PAI [19, 20]. Conversely, Addison’s disease is a rare APS complication. Only 0.4% of patients with APS develop PAI over a follow-up period of 5 years [21]. When catastrophic APS occurs, the frequency of adrenal insufficiency (AI) unsurprisingly rises to 10–26% [22, 23].

PAI may sometimes be the first clinical manifestation of APS and is the most common endocrine complication [24]. The mortality rate of patients with APS complicated by AI is 3.81%, a rather high percentage given their young age [25]. The presumable pathogenetic substratum of hypoadrenalism is thrombosis of the adrenal veins with consequent hemorrhagic infarction [26].

This article reports the case of a young female patient with a history of SLE and APS who presented with an acute adrenal crisis. PAI secondary to adrenal hemorrhage was unmasked during a stressful situation triggered by inflammation. We have also conducted a literature review to identify similar cases.

## Methods

We performed a computer-assisted search to identify isolated case reports, retrospective studies, and reviews in the literature associating AI with APS and/or SLE published until November 2022, using the PubMed, Google Scholar, Science direct, and Springer Link databases. The main keywords used were Addison’s disease, adrenal insufficiency, adrenal failure, hypoadrenalism, adrenal hemorrhage, adrenal infarction, antiphospholipid syndrome, and systemic lupus erythematosus. We excluded cases where the cause of adrenal insufficiency was obviously irrelevant despite the presence of the above autoimmune disorders and articles written in languages other than English.

## Case presentation

A 30-year-old female was admitted to the emergency department (ED) with fever, lethargy, and syncopal episodes within the past 48 h. She had a 2-year medical history of SLE, initially manifested by myocarditis-induced cardiopulmonary arrest and secondary APS with recurrent episodes of unprovoked deep venous thrombosis (DVT) and sub-segmental pulmonary embolism. Regarding her family history, her parents were diagnosed with type 2 diabetes mellitus and her sister with idiopathic thrombocytopenic purpura.

The patient was regularly treated with hydroxychloroquine, mycophenolic acid, bisoprolol, and coumarin anticoagulant, until she recently switched to tinzaparin after a surgical operation. She was compliant with medication, but she admitted that, formerly, while treated with acenocoumarol, international normalized ratio (INR) values often remained below the predetermined range of 2–3.

**Fig. 1:**
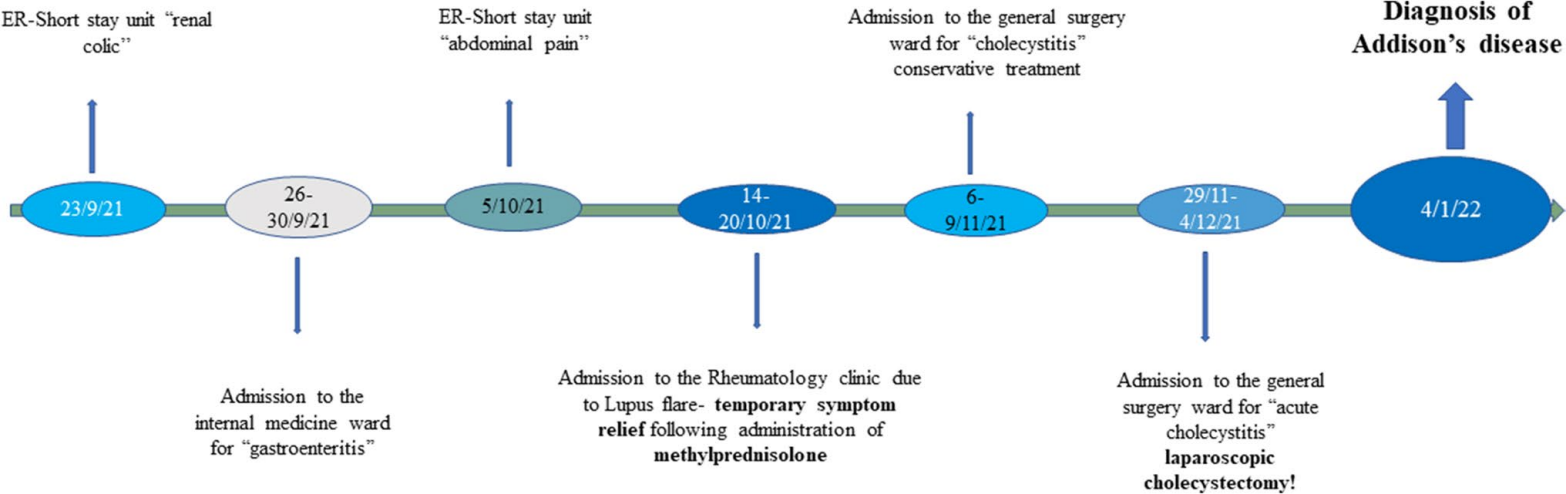
Multiple admissions of the patient due to PAI-related symptoms misdiagnosed for other conditions

The patient presented with diffuse abdominal pain, general weakness, arthralgias, hyperpigmentation, nausea, vomiting, anorexia and unintentional weight loss of 48 kg over a 5-month-period. She complained of watery diarrhea for 4 weeks and secondary amenorrhea for the last 3 months. Her clinical status deteriorated progressively but was overlooked by health care professionals who repeatedly evaluated her. Of note, clinical manifestations were temporarily ameliorated 2.5 months ago after methylprednisolone administration for a lupus flare. One month prior to current admission, the patient’s intense gastrointestinal symptoms baffled the surgeons who attributed them to cholelithiasis, proceeding to laparoscopic cholecystectomy. After surgery, although the abdominal pain was slightly relieved, the rest of the symptoms persisted and deteriorated (Fig. 1).

**Fig. 2:**
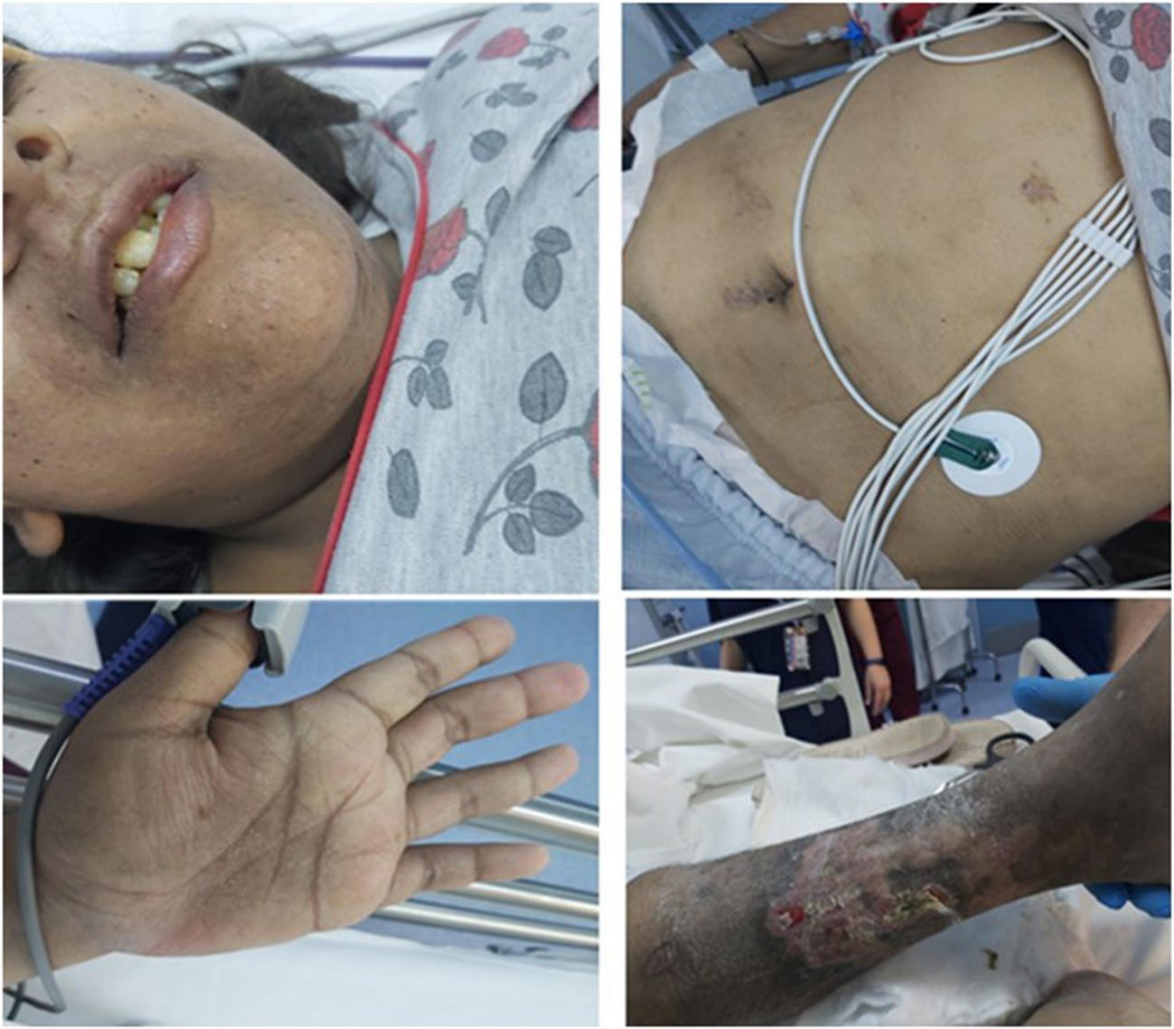
Diffuse skin hyperpigmentation more conspicuous in palmar creases and scars after laparoscopic cholecystectomy. An ulcer on the left lower extremity (The patient gave consent to the hospital for the use of pictures.)

In the ED, the patient was found to be confused (Glasgow Coma Scale: 12/15), tachypneic, hypoxemic (partial pressure of oxygen pO2 = 77 mmHg), anuric, and hemodynamically unstable with hypotension (79/38 mm Hg) and tachycardia (130 beats per min). Mild epigastric tenderness on palpation, severe dehydration, and generalized skin hyperpigmentation were noted on physical examination. The areas most pigmented were the palmar creases, axillae, areolae, abdominal scars, and oral mucosa. An additional physical finding was an ulcer on the left lower extremity (Fig. 2).

Initial laboratory investigations revealed anemia, severe electrolyte dysregulation, increased inflammatory markers, acute renal failure, and metabolic acidosis. Urinalysis indicated pyuria and urine sediment microscopy was suggestive of a urinary tract infection and lupus nephritis (Table 1).

**Table 1:** Summary of hematochemical, endocrine, and immunologic findings

	Values	Normal range
RBC	**3.38**	3.8–5.3 M/μL
HGB	**9.8**	12–16 g/dl
HCT	**29.8**	37–47%
MCV	88.1	80–99 fL
MCH	29.1	27–32 pg
MCHC	33	32–35 g/dl
Anti-Xa	0.2	Prophylactic range: 0.2–0.5 IU/ml Therapeutic range: 0.5–1.2 IU/ml
D-dimer	0.33	0–0.55 mg/l
aPTT	**40.5**	25.9–36.6 s
PT	**20.5**	10.4–14 s
INR	**1.75**	0.85–1.2
Urea	**92**	17–43 mg/dl
Creatinine	**5.84**	0.55–1.02 mg/dl
Sodium	**125**	136–146 mEq/l
Potassium	**6.1**	3.5–5.1 mEq/l
Calcium (corrected)	**10.94**	8.8–10.6 mg/dl
Magnesium	**1.5**	1.9–2.5 mg/dl
hs Troponin I	**1916.6**	< 11.6 pg/ml
Total cholesterol	**51**	< 200 mg/dl
LDL cholesterol	**5**	< 100 mg/dl
HDL cholesterol	**14**	> 40 mg/dl
CRP	**3.73**	< 0.5 mg/dl
ESR	**95**	1–25 mm/1^st^ hr
Procalcitonin	**1.03**	< 0.05 ng/ml
Cortisol	**1**	3.5–19 μg/dl
Aldosterone	**21.5**	29.4–161.5 pg/ml
DHEAS	**5,7**	95.8–511.7 μg/dl
Androstenedione	**0.09**	0.25–3.44 ng/ml
17-ΟΗ-progesterone	0.48	0.13–1.67 ng/ml
Testosterone	< 0,13	0.09–1.3 ng/ml
SHBG	46	18–114 nmol/l
C3 complement	**45.5**	87–187 mg/dl
C4 complement	**4.96**	15–47 mg/dl
ANA	**1:1280 diffuse**	U/ml
ENA	**1.22**	> 1 positive
Anti-ds-DNA	**22.1**	> 18 U/ml positive
Anti-β2-GP1 IgM	**39.4**	> 18 U/ml positive
Anti-β2-GP1 IgG	8.22	> 18 U/ml positive
aCL IgM	**54.4**	> 18 MPL/ml positive
aCL IgG	15.5	> 18 MPL/ml positive
LA	**positive**	
Direct Coombs	**positive**	
Anti-adrenal	negative	< 1:20 negative

Entries in bold indicate abnormal values

An acute adrenal crisis was suspected particularly based on hyponatremia, hyperkalemia, renal failure, hypovolemic shock, confusion, and hyperpigmentation. The crisis was then confirmed by the detection of a markedly decreased serum cortisol (1 μg/dl) under stressful conditions. Low aldosterone, androstenedione, dehydroepiandrosterone sulfate (DHEAS), and testosterone levels indicated impaired mineralocorticoid and androgen production.

The patient was immediately treated with intravenous hydrocortisone 100 mg as a bolus, followed by continuous infusion at a rate of 10 mg/h and fluid resuscitation. Mental status improved rapidly. Blood pressure responded but inadequately; therefore, vasopressor support with norepinephrine was ultimately required. The patient was subsequently admitted to the intensive care unit (ICU) due to hemodynamic compromise, indications of shock-induced cardiac ischemia and/or recurrence of lupus myocarditis, and acute renal failure. Within a few hours from admission, she became febrile (peak temperature 38.7 ^◦^C). The patient was treated with antibiotics for an Enterococcus gallinarum and Escherichia coli urinary tract infection. She was also treated with continuous hydrocortisone infusion, crystalloid fluids, red blood cell transfusion, calcium gluconate, vasoactive agents, antiarrhythmics, and low-molecular-weight heparin (LMWH) at a therapeutic dosage along with supplemental oxygen.

The fever ultimately resolved on the second day of ICU admission. On the fourth day, leukopenia and thrombocytopenia appeared on the complete blood count (CBC) panel, which resolved 3 days later. All electrolyte disturbances were reversed after the first 2 days in the ICU. Troponin I exhibited a peak concentration in the first 24 h and then decreased slowly. Serum creatinine and CRP declined and eventually normalized by the sixth day of hospitalization. Overall symptoms improved and vital signs stabilized within 48 h. Vasopressor and oxygen therapy were withdrawn, and urine output exceeded 1 ml/kg/h. Due to heart failure, the patient was commenced on furosemide.

Three days after ICU admission, she was transferred to the endocrine clinic for further management. Intravenous hydrocortisone was gradually tapered in line with her clinical response, with close monitoring of her blood pressure, symptoms, and laboratory tests. She was then switched to oral steroid therapy. The pituitary hormonal evaluation demonstrated no other hormonal deficits. To identify the etiology of primary adrenal insufficiency, autoantibodies to the adrenal cortex were requested, which were negative.

**Fig. 3:**
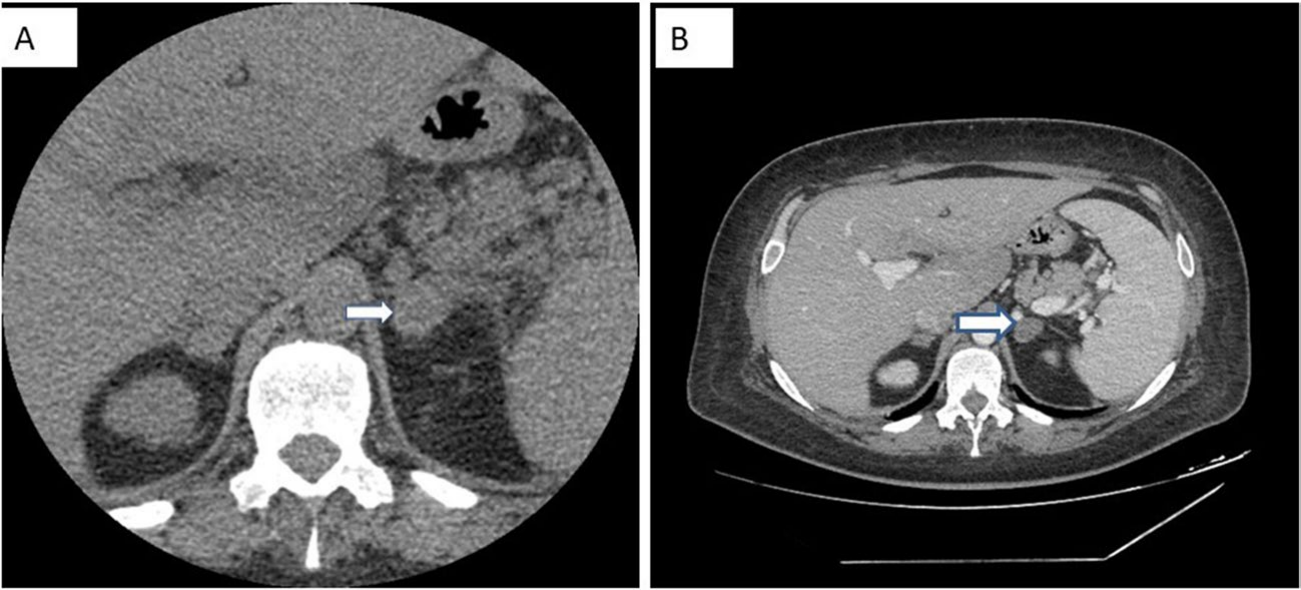
**A** Adrenal computed tomography (CT) showing bilateral enlargement of the adrenal glands mainly of the left, with intermediate mean density, punctate calcifications, and perinephric fluid, consistent with a recent bilateral adrenal hemorrhage. **B** Repeat adrenal CT scan showing a decrease in size of both adrenal glands, suggesting remission of the hemorrhage

Adrenal computed tomography (CT) scan performed on the seventh day of hospitalization revealed bilateral enlargement of the adrenal glands (the left measuring 2.55 cm and the right 1.75 cm) with intermediate mean density (35 Hounsfield Units), punctate calcifications on the left adrenal gland and perinephric fluid. Contrast medium was not administered to protect renal function. Overall features were consistent with a subacute bilateral adrenal hemorrhage (Fig. 3A). A repeat adrenal CT scan 9 days later showed a decrease in size of both adrenal glands (Fig. 3B), suggesting remission of the hemorrhage. A chest CT scan depicted lymph nodes which were stable in size and number, compared with those detected on previous imaging 2 years earlier. Mild bilateral pleural effusion and ground glass opacities were noted.

The patient was subsequently transferred to the rheumatology clinic due to clinical and biochemical evidence of active SLE. Accordingly, methylprednisolone, mycophenolic acid, and hydroxychloroquine were administered (Table 1). Finally, the patient was safely discharged from the hospital after 18 days. Upon dismissal, the results of the laboratory tests were found to be normal except for mild anemia (Hb: 11.3 g/dl). Due to the thrombotic APS, she received counseling about the need for close INR monitoring in the setting of bridging therapy of LMWH with acenocoumarol with a target INR of 2,5-3 to avoid future thrombotic events. Methylprednisolone was slowly tapered to maintenance doses of hydrocortisone and she was instructed to start fludrocortisone as soon as the daily hydrocortisone dose was below 50 mg, since this dose is equivalent to 0,1 mg fludrocortisone. The rest of her medication consisted of mycophenolic acid, hydroxychloroquine, bisoprolol, esomeprazole, cholecalciferol, calcium carbonate, and magnesium.

To prevent a new adrenal crisis, the patient was educated regarding hydrocortisone dose adjustments during intercurrent illness or other stress conditions, and she was also provided with a steroid emergency card and hydrocortisone emergency kit. Good clinical outcome was confirmed at a follow-up appointment 2 months later. She continued replacement therapy and reported improvement in energy levels and appetite, weight gain, and regular menstruation. Hyperpigmentation began to subside and blood pressure was normal without postural hypotension. Blood testing showed normal hemoglobin concentration, serum electrolyte levels, and renal function. She is currently an outpatient and has regular endocrinology, rheumatology, and cardiology follow-up consults.

## Discussion

In this case report, we have presented a 30-year-old female patient with a history of APS secondary to SLE who developed bilateral adrenal hemorrhagic infarction and consequently PAI. PAI is a disorder characterized by the decreased production or action of glucocorticoids with concomitant deficiency in mineralocorticoids and/or adrenal androgens, which may result in acute and potentially life-threatening adrenal crisis [16]. Clinical features vary depending on the rate and extent of adrenal function loss and can be nonspecific such as fatigue, weight loss, anorexia, abdominal pain, nausea, vomiting, diarrhea, or constipation, myalgias, arthralgias, and neuropsychiatric symptoms [7, 27, 28]. Hypotension accompanied by orthostatic symptoms and salt craving are signs of mineralocorticoid deficiency. Females may notice loss of axillary and public hair, reduced energy, and decreased libido because of decreased adrenal androgen secretion. Oligo-amenorrhea may be due to chronic illness, weight loss, or autoimmune primary ovarian insufficiency [7]. In cases of autoimmune adrenalitis, signs of autoimmune comorbidities often coexist [29]. Hyperpigmentation, which is caused by the excessive secretion of pro-opiomelanocortin (POMC), a prohormone that is cleaved into the biologically active hormones, ACTH and α-melanocyte stimulating hormone (α-MSH), is most conspicuous in areas subject to sun exposure and mechanical friction [30].

Biochemically, hyponatremia and hyperkalemia are typical; additional findings such as hypercalcemia, azotemia, anemia, eosinophilia, and hypoglycemia can also coexist [18]. The diagnosis can be confirmed by a low morning cortisol serum concentration in combination with elevated ACTH (usually more than two-fold the upper reference limit). Low aldosterone along with high renin concentration/plasma renin activity and low DHEAS are helpful indications of PAI [7]. The “gold standard” for the diagnosis of PAI is currently the short Synacthen test, which may be performed only when the patient is stable. A peak cortisol concentration above 18 µg/dl at 30 or 60 min is widely accepted as a normal response [31].

Conventional glucocorticoid substitution with hydrocortisone is generally effective but still has pharmacological limitations [32]. Dual-release hydrocortisone, a formulation developed to obtain a more physiological circadian-based serum cortisol exposure-time profile, enables once-daily dosing of hydrocortisone [33]. Modified-release hydrocortisone has a delayed and sustained absorption profile [34, 35]. Continuous subcutaneous hydrocortisone infusion using pumps is the most promising approach [36]. If deficit of mineralocorticoids is confirmed, patients should receive fludrocortisone at a single dose of 0.05–0.2 mg/d [37]. Despite otherwise optimized replacement therapy with glucocorticoids and mineralocorticoids, quality of life may remain impaired. A trial of DHEA (25–50 mg/d) is suggested in women with PAI complaining of low libido, depressive symptoms, and reduced energy levels [7].

Primary adrenal insufficiency and adrenal crisis rarely manifest in patients with an underlying autoimmune disease such as SLE and APS [38, 39]. SLE is a connective-tissue disorder characterized by the loss of self-tolerance, production of autoantibodies against nuclear and cytoplasmic antigens, and immune complex deposition. Its clinical presentation is heterogeneous, with a relapsing and remitting course, and may involve one or more systems [40]. The EULAR/ACR classification criteria include positive ANA at least once as an obligatory criterion, followed by additive weighted criteria grouped in seven clinical (constitutional, hematological, neuropsychiatric, mucocutaneous, serosal, musculoskeletal, and renal) and three immunological domains [(aPL, SLE-specific antibodies (anti-dsDNA, or anti-Smith), low complement proteins]. Each criterion is assigned a weight of 2 to 10. If a patient accumulates ≥ 10 points and at least one clinical criterion is fulfilled, the disease is classified as SLE [41].

The antiphospholipid syndrome is a systemic disease representing one of the most common hypercoagulation states. It can occur either as a primary condition (primary APS) or in association with SLE or other autoimmune disease (secondary APS). The most severe form of APS is an accelerated form of APS resulting in multiorgan failure [42]. APS has a wide spectrum of vascular and obstetric manifestations associated with thrombotic and inflammatory mechanisms orchestrated by aPL antibodies [43]. To fulfil the revised Sapporo criteria for APS, at least one clinical criterion should be present and one or more types of aPL antibodies should be detected on two consecutive occasions at least 12 weeks apart. aPL antibodies are an expanding group of autoantibodies directed against anionic phospholipids and phospholipid-associated plasma proteins. The aPL detection tests are aCL antibody (IgG or IgM) enzyme-linked immunosorbent assay (ELISA), anti-β2-GP1 antibody (IgG or IgM) ELISA, and LA assay [44]. Triple positivity for LA, aCL, and anti-β2-GP1 is associated with an increased risk for the first thrombotic or obstetric event in previously healthy individuals and a high rate of recurrences in patients receiving antithrombotic treatment [45]. Endocrine complications are undoubtedly very rare. Bilateral adrenal hemorrhage is such a clinical manifestation that may lead to PAI [24, 46–48].

Cases of thrombotic APS, including adrenal hemorrhagic infarction, should be treated with long-term anticoagulation therapy. Treatment with vitamin K antagonists (VKAs), either coumarin anticoagulant or warfarin, is the cornerstone of secondary prevention of thromboembolic events [43]. According to EULAR recommendations, patients with recurrent venous thromboses despite treatment with VKA and a target INR of 2–3 should be evaluated for the intensity of anticoagulation, adherence to therapy, and to INR monitoring. If an INR of 2–3 is achieved, the addition of low-dose aspirin, an increase of INR target to 3–4, or a switch to LMWH may be considered [43].

To date there have been several isolated case reports, retrospective studies, and reviews in the literature associating AI with APS and SLE. The number of cases referring to AI in the setting of SLE or APS, published until 10 September, 2017, was 97, according to Lee et al. [25]. The remaining publications identified from October 2017 to November 2022 are 12, including 14 subjects, the basic characteristics of whom are presented in Table 3. We estimate that the total number of existing cases in the literature approximates 111.

Information about pathogenesis, diagnosis, and therapeutic approaches of this rare condition was derived principally from three studies (Table 2). The first one, carried out by Espinosa and colleagues in 2003, was a review of 80 cases from the literature and six from their own cohort with APS (primary or secondary to SLE) and adrenal involvement. The authors concluded that the close relationship between APS and hypoadrenalism was more than coincidental [49]. The second review was conducted by Lee et al. who studied publications until 2017 including 97 subjects who experienced AI related to APS or SLE [25]. The third study by Ramon et al. was a retrospective study of 16 patients with bilateral adrenal hemorrhage or infarction secondary to APS seen in Pitié-Salpêtrière Hospital between 1990 and 2010 [50].

**Table 2:** Clinical and laboratory findings of AI in our case and their frequency in similar cases reported in review references

Symptoms-laboratory and imaging findings	Current case	Lee et al. [25]	Espinosa et al. [49]	Ramon et al. [50]
Peak age at diagnosis of AI	30 yrs	40–49 yrs	43 yrs	33.5 yrs
Dx of AI post SLE and/or APS (%)	yes	62	na	69
Deep vein thrombosis (%)	yes	25.71	na	62.5
Pulmonary embolism (%)	yes	7.61	na	37.5
Weight loss (%)	yes	13.31	13	na
General weakness (%)	yes	9.52	41	na
Fatigue (%)	yes	6,62	na	na
Lethargy (%)	yes	6.67	19	na
Dizziness (%)	yes	3.81	na	na
Fever (%)	no	33.3	40	na
Pigmentation (%)	yes	6.67	10	na
Abdominal pain (%)	yes	39.04	55	na
Hypotension (%)	yes	7.61	54	na
Nausea(%) Vomiting(%) Diarrhea (%) Basal cortisol (< 3 μg/dL) (%) High ACTH(%) Low Aldosterone (%) Hyponatremia(%) Hyperkalemia (%) Anti-cardiolipin antibody (%) Lupus anticoagulant (%) Adrenal hemorrhage (%)	yes yes yes na yes yes yes yes yes yes yes	19.05 23.81 3.81 68 84 100 50.48 27.62 53.3 45.71 34	na 31 4 96 98 na 80 80 90 95 27	na na na 81.25 83.3 50 na na 100 87 25

*AI* adrenal insufficiency, *APS* antiphospholipid syndrome, *SLE* systemic lupus erythematosus, *yrs* years, *Dx* diagnosis, *na* not available

Among the mechanisms proposed to explain the pathogenesis of PAI, the most cited one is based upon the unique vascular anatomy of the adrenal glands. The imbalance between the rich arterial supply and the limited drainage by a single adrenal vein predisposes to venous thrombosis, especially in the context of APS. The adrenal gland is supplied by the superior, middle, and inferior adrenal arteries, which originate from the inferior phrenic, the abdominal aorta, and the renal artery, respectively, and then branch out to form smaller arteries. In the zona reticularis, a capillary plexus is formed which drains into the medullary sinusoids and eventually into the large central vein [51]. The transition from the arterial to the capillary system is so abrupt that it constitutes a “vascular dam” which causes the accumulation and stasis of blood. Moreover, the eccentric muscular arrangement of the adrenal veins makes them vulnerable to the formation of thrombi in pockets of turbulence and local stasis when the bundles contract [26]. These factors predispose to adrenal vein thrombosis, usually bilateral, with disruption of adrenal gland outflow that ultimately leads to hemorrhagic infarction and hypoadrenalism.

A recent concept linked the development of PAI to the high density of late endosomes in cells of the zona fasciculata. These are organelles which participate in cholesterol trafficking and protein sorting. Their membranes contain lysobisphosphatidic acid, a target of aPL antibodies. The antibodies promote apoptosis and release of lysosomal proteinases, which in turn activate endothelial cells, leading to microthrombosis [52].

Τhe peak incidence of AI related to APS or SLE (or both) is observed in the fifth decade of life (23.81%), with a relatively high frequency between 30 and 39 years (20.95%). The prevalence of adrenal involvement is slightly higher in male APS patients (53.3%) [25]. Regarding past medical history, 37.14% of patients had former thrombotic events, mainly deep vein thrombosis and pulmonary embolism. Abortion and miscarriage (10.48%) were the predominant past obstetric manifestations [25]. Similarly, in the study conducted by Espinosa et al., 36% of patients diagnosed with PAI had no previous APS complications, 40% had already deep venous thrombosis, 17% pulmonary embolism, and 19% arterial occlusions, while 18% of females reported a history of spontaneous fetal losses [49]. Ramon et al. also showed that PAI was the first manifestation of APS in one-third of patients, whereas the rest fulfilled prior clinical criteria of APS. The author estimates that the mean time interval between diagnosis of APS and onset of adrenal involvement is 5.1 years [50].

In the systematic review by Lee et al., the principal presenting symptoms of AI in the context of APS or SLE were abdominal pain, fever, and vomiting [25]. Hyponatremia was the leading finding in 77.94%, while hyperkalemia was present in half of the patients. ACTH was above the normal range in 84% of cases and aldosterone was low in all. aCL were positive in 59.57%, LA in 51.06%, and ANA in 31.91% of cases [25]. Espinosa et al. noted that clinical presentation typically involved abdominal pain, hypotension, fever, nausea, vomiting, and weakness [49]. Hyponatremia, often accompanied by hyperkalemia, was present in most patients. Cortisol levels were low and ACTH levels high in nearly all cases. LA was detected in 97% and aCL were positive in 93%, whereas simultaneous presence of the above aPL antibodies was found in 89% of patients. Hypocomplementemia was documented in one-third of cases (Table 2) [49].

In the past, many cases of adrenal hemorrhage/infarction were diagnosed post-mortem. Currently, CT and MRI contribute significantly to the investigation of adrenal pathology. An adrenal hemorrhage appears on imaging in 57% of cases with APS and adrenal involvement. The second most common finding is adrenal infarction (14%), usually with a hemorrhagic component. Histopathologic studies show hemorrhagic infarction with venous thrombosis (55%) and adrenal hemorrhage (27%) [49].

In the case we have reported, PAI was confirmed before imaging. CT scan performed showed bilateral adrenal enlargement attributed to recent adrenal hemorrhage, consistent with many cases in the literature. Since the workup for other recognizable causes was negative, bilateral adrenal hemorrhagic infarction in the context of APS was deemed the most likely cause of PAI. We speculate that the precipitating factor was the subtherapeutic dose of anticoagulants for the APS. Specifically, anti-Xa levels on admission were at the lowest limit of prophylactic range while the patient was on LMWH therapy. Furthermore, when she was previously treated with acenocoumarol, the INR target was not achieved. A contributing factor to the development of the adrenal crisis was presumably the recent occurrence of bacterial urinary tract infection.

In our case, PAI manifested as an acute adrenal crisis 2 years after the first APS-related episodes, namely, deep venous thrombosis and pulmonary embolism. Adrenal failure had probably evolved slowly over at least the last 5 months since clinical symptoms and electrolytic disturbances of hyponatremia and hyperkalemia had been recorded at previous visits to the ED (Fig. 1). Except for the urinary infection as a significant stress condition that led to AI development, the cholecystectomy 1 month prior most probably contributed to the AI and was, moreover, unnecessary considering that presenting symptoms did not improve but rather deteriorated. Clinical, laboratory, and imaging findings in our patient and their frequency in other similar reported cases are shown in Table 3.

**Table 3:** Clinical and laboratory findings of AI in similar case reports over the last 5 years

Symptoms-laboratory and imaging findings	Chu et al. (2017) [53]	Oliveira et al. (2018) [47]	Kolinioti et al. (1^st^ case 2018) [54]	Kolinioti et al. (2^nd^ case) [54]	Bansal et al. (2019) [19]	You et al. (2019) [55]	Warriach et al. (2020) [56]	Kozamernik et al. (1st case 2020) [48]	Kozamernik et al (2^nd^ case) [48]	Medina et al. (2020) [57]	Arosemena et al. (2020) [58]	Yazdi et al. (2021) [59]	Billet et al. (2022) [60]	Tan et al. (2022) [61]
Age at diagnosis of AI (yrs)	35	36	65	72	50	50	64	27	54	36	46	32	54	45
Dx of AI post SLE and/or APS	no	yes	no	no	no	yes	yes	no	yes	yes	yes	no	yes	no
Deep vein thrombosis	yes	yes	yes	yes	yes	yes	yes	no	yes	yes	yes	yes	yes	yes
Pulmonary embolism	yes	no	no	yes	yes	no	no	yes	no	yes	yes	no	no	yes
Weight loss	na	na	na	na	na	na	no	na	na	yes	yes	na	na	na
General weakness	yes	yes	yes	yes	na	na	no	na	na	na	yes	na	no	no
Fatigue	yes	yes	yes	yes	na	na	yes	na	na	na	yes	na	yes	no
Lethargy	no	no	yes	yes	no	no	no	no	no	no	yes	no	no	no
Dizziness	no	na	na	na	no	no	yes	na	na	na	yes	na	no	no
Fever	yes	no	yes	yes	yes	yes	na	yes	yes	no	no	no	no	no
Pigmentation	yes	no	no	no	no	no	no	no	yes	yes	no	yes	no	no
Abdominal pain	yes	no	yes	yes	yes	yes	yes	no	no	no	yes	yes	no	yes
Hypotension	yes	no	yes	yes	yes	no	yes	na	no	yes	yes	yes	yes	no
Nausea	na	yes	no	no	no	yes	yes	no	yes	no	no	no	yes	no
Vomiting	na	yes	no	no	no	yes	yes	no	yes	no	no	no	yes	no
Diarrhea	no	no	no	no	no	no	no	yes	no	no	no	no	no	no
Basal cortisol (< 3 μg/dl)	no	yes	no	na	no	yes	no	yes	yes	no	na	no	yes	yes
High ACTH	yes	yes	yes	na	yes	yes	yes	yes	yes	yes	na	yes	yes	yes
Low aldosterone	na	yes	na	na	no	no	na	yes	yes	na	na	no	no	yes
Hyponatremia	yes	yes	yes	yes	no	yes	yes	yes	yes	yes	yes	no	yes	na
Hyperkalemia	yes	yes	no	no	no	no	yes	yes	yes	no	no	no	no	na
Anti-cardiolipin antibody	yes	na	yes	yes	yes	na	yes	na	na	yes	yes	yes	na	yes
Lupus anticoagulant	yes	na	na	na	na	na	na	na	na	na	na	na	na	yes
Adrenal hemorrhage	yes	yes	yes	yes	yes	yes	yes	yes	yes	yes	yes	yes	yes	yes

*AI* adrenal insufficiency, *APS* antiphospholipid syndrome, *SLE* systemic lupus erythematosus, *yrs* years, *Dx* diagnosis, *na* not available

The recommended treatment of APS patients with adrenal involvement includes mainly hormonal replacement with glucocorticoids and mineralocorticoids besides anticoagulation therapy. Hydrocortisone was the first-line steroid replacement in 40% of cases analyzed by Lee et al., followed by fludrocortisone and prednisolone. Volume replacement was initiated in 12% of patients. Anticoagulants mostly administered were warfarin (23%) and heparin (14%) [25].

Data on the prognosis of APS-related Addison’s disease is scarce. Reassessment usually shows complete, irreversible adrenal failure and shrinkage of the adrenal glands, with the need for lifelong replacement therapy. Repeated Synacthen tests demonstrate poor cortisol response in 80% of patients but partial recovery of adrenal function is sporadically observed [50].

## Conclusion

PAI is very rare in patients with SLE, but reports of the association suggest the presence of APS in these patients. PAI can be the first manifestation or may develop later during APS. Due to its insidious onset and non-specific symptoms, physicians may overlook PAI. As a result, the diagnosis is often delayed and formulated only in the presence of an acute adrenal crisis.

A high index of clinical suspicion is required in aPL-positive patients who present with undue abdominal pain, weakness, hypotension, fever, nausea, hyperpigmentation, hyponatremia, or hyperkalemia. If an adrenal crisis is suspected, blood pressure and fluid balance status should be assessed and appropriate laboratory investigations, including paired cortisol and ACTH, should be promptly performed. Steroid treatment should be initiated promptly without the delay of awaiting results. Conversely, in all cases of PAI associated with imaging findings of bilateral adrenal enlargement and hemorrhage, screening for aPL is imperative.
